# Bioequivalence Study of Two Orodispersible Rizatriptan Formulations of 10 mg in Healthy Volunteers

**DOI:** 10.3390/scipharm84030514

**Published:** 2016-06-13

**Authors:** Mercè Cánovas, Francisco Polonio, Francesc Cabré

**Affiliations:** Laboratorios Lesvi, S.L.-Invent Farma Group, Sant Joan Despí, Barcelona 08970, Spain; fpolonio@lesvi.com (F.P.); fcabre@lesvi.com (F.C.)

**Keywords:** Antimigraine agent, rizatriptan, bioequivalence, pharmacokinetics

## Abstract

The aim of the study was to assess the bioequivalence and tolerability of two different oral formulations of rizatriptan. A bioequivalence study was carried out in 40 healthy volunteers according to an open label, randomized, two-period, two-sequence, crossover, single dose, and fasting conditions design. The test and reference formulations were administered in two treatment days, separated by a washout period of seven days. Plasma concentrations of rizatriptan were obtained by the LC/MS/MS (Liquid chromatography tandem-mass spectrometry) method. Log-transformed AUC_0-t_ (area under the plasma concentration-time curve from zero to the last measurable concentration) and C_max_ (maximum plasma concentration) values were tested for bioequivalence based on the ratios of the geometric means (test/reference). The t_max_ (time to reach maximum plasma concentration) was analysed nonparametrically. The 90% confidence intervals of the geometric mean values for the test/reference ratios for AUC_0-t_ and C_max_ were within the bioequivalence acceptance range of 80%–125%. According to the European Guideline, it may therefore be concluded that the test formulation of rizatriptan 10 mg orodispersible tablet is bioequivalent to the reference formulation (Maxalt^®^ Max 10 mg oral lyophilisate). The safety profile of both formulations was consistent with the summary of the product characteristics.

## 1. Introduction

A migraine is the most common headache disorder encountered in clinical practice. Approximately 25% of women and 9% of men are affected by this disorder. More than half of these patients complain of severe or very severe headache and of significant limitations of their social and work activities, with some confined to bed by the attacks [1].

It is generally recognized that the primary cause of migraine headaches lies in the brain and may be related to cortical hyperexcitability and brainstem nuclei dysfunction. Activation of the trigeminovascular system leads to release of vasoactive neuropeptides, resulting in vasodilation of the meningeal vessels and neurogenic inflammation. Both peripheral and central sensitizations have a role in migraine pain. The International Classification of Headache Disorders (ICHD-II) describes migraine as a recurrent headache disorder manifesting in attacks lasting 4–72 h. Typical characteristics of the headache are unilateral location, pulsating quality, moderate or severe intensity, aggravation by routine physical activity, and association with nausea and/or photophobia and phonophobia [2].

The objective of acute migraine therapy is to restore the patient’s ability to function by rapidly and consistently alleviating the head pain and the associated symptoms. The introduction of triptans into the market in 1993 increased the therapeutic options in migraine patients considerably. Triptans are effective drugs for the acute treatment of migraines, and are the drugs of choice for disabling migraine attacks. The currently available triptans show many similar characteristics, but there are also some clinically relevant pharmacological differences. Amongst the triptans, oral rizatriptan has been reported to be more effective than sumatriptan, naratriptan, and zolmitriptan. In a crossover study, rizatriptan was found to be superior to the ergotamine and caffeine combination in relieving acute migraine attacks [3,4,5,6].

Rizatriptan binds selectively with high affinity to human 5-HT_1B_ (5-hydroxytryptamine) and 5-HT_1D_ receptors and has little or no effect or pharmacological activity at 5-HT_2_, 5-HT_3_; adrenergic α_1_, α_2_, or β; D_1_, D_2_, dopaminergic, histaminic H_1_; muscarinic; or benzodiazepine receptors. The therapeutic activity of rizatriptan in treating migraine headaches may be attributed to its agonist effects at 5-HT_1B_ and 5-HT_1D_ receptors on the extracerebral intracranial blood vessels that are thought to become dilated during an attack and on the trigeminal sensory nerves that innervate them. Activation of these 5-HT_1B_ and 5-HT_1D_ receptors may result in constriction of pain-producing intracranial blood vessels and inhibition of neuropeptide release that leads to decreased inflammation in sensitive tissues and reduced central trigeminal pain signal transmission [7].

Rizatriptan is rapidly and completely absorbed following oral administration. The mean oral bioavailability of the oral lyophilisate is approximately 40%–45%, and mean peak plasma concentrations (C_max_) are reached in approximately 1.58 h. The time to reach maximum plasma concentration (t_max_) following administration of rizatriptan as the oral lyophilisate formulation is delayed by 30–60 min relative to the tablet. Rizatriptan is minimally bound (14%) to plasma proteins. The volume of distribution is approximately 140 litres in male subjects, and 110 litres in female subjects. The primary route of rizatriptan metabolism is via oxidative deamination by monoamine oxidase-A (MAO-A) to the indole acetic acid metabolite, which is not pharmacologically active. *N*-monodesmethyl-rizatriptan, a metabolite with activity similar to that of the parent compound at the 5-HT_1B/1D_ receptors, is formed to a minor degree, but does not contribute significantly to the pharmacodynamic activity of rizatriptan. The plasma half-life of rizatriptan in males and females averages 2–3 h. The plasma clearance of rizatriptan averages about 1000–1500 mL/min in males and about 900–1100 mL/min in females; about 20%–30% of this is renal clearance. Consistent with its first pass metabolism, approximately 14% of an oral dose is excreted in urine as unchanged rizatriptan, while 51% is excreted as indole acetic acid metabolite. No more than 1% is excreted in urine as the active N-monodesmethyl metabolite. If rizatriptan is administered according to the maximum dosage regimen, no drug accumulation in the plasma occurs from day-to-day [7].

According to the European Guideline [8], two medicinal products containing the same active substance are considered bioequivalent if they are pharmaceutically equivalent, or pharmaceutical alternatives and their bioavailabilities (rate and extent) after administration in the same molar dose lie within acceptable predefined limits. The aim of the study reported here was to compare the rate and extent of absorption of two oral formulations of rizatriptan 10 mg (test: orodispersible tablet, reference: oral lyophilisate) in a single dose, two-period, two-sequence, crossover, and randomised study in healthy volunteers. As a secondary objective the safety of both formulations was assessed.

## 2. Materials and Methods

### 2.1. Study Design 

The study was conducted in a single dose, two-period, two-sequence, fasting, open label, crossover randomised design, comparing the bioavailability of a new generic formulation (test) and a trade name formulation (reference) of rizatriptan 10 mg.

During each period, the volunteers were admitted to the Phase I Unit. After an overnight fasting period of at least 8 h, they received one 10 mg rizatriptan orodispersible tablet (test) or one 10 mg rizatriptan oral lyophilisate (reference) formulation according to the randomisation scheme. Each volunteer was assigned randomly to one of the sequences. The medication was placed on the tongue (without swallowing) after wetting the mouth with 20 mL of plain water. Each subject was asked to allow the tablet to orally dissolve and not to chew until completely disintegrated. The resulting suspension was swallowed. No water was provided.

During the hospitalization (until +24 h post-dose), caloric and liquid intake were controlled and standardized by the investigator’s team. Following drug administration, study subjects continued in fasting conditions for a minimum of 4 h and standard meals were served at scheduled times (+4 h, +8 h, and +13 h after drug administration). No fluid intake was allowed from 1 h before until 2 h after drug administration.

In the second period, the volunteers received the alternate product (test or reference) after a wash-out period of one week.

Blood samples (3 mL each) were collected into polypropylene tubes treated with K_2_EDTA (dipotassium ethylenediaminetetraacetic acid) at pre-dose (baseline), 0.25, 0.50, 0.75, 1.00, 1.25, 1.50, 1.75, 2.00, 2.25, 2.50, 2.75, 3.00, 4.00, 6.00, 8.00, 10.00, 12.00, and 24.00 h post-dosing in each period. Following centrifugation (3000 rpm, 4 °C, 10 min), the plasma supernatant was aliquoted into two polypropylene tubes and stored frozen at −20 °C until assayed.

### 2.2. Study Subjects

Subjects included in the study were healthy female and male volunteers ranging from 20 to 32 years old (mean ± SD; 24.2 ± 2.7), weigthing 47.5–97.6 kg (69.2 ± 13.9), with a height between 156 and 190 cm (172.3 ± 9.7) and a Body Mass Index (BMI) between 19.1 and 29.1 kg/m^2^ (23.1 ± 2.8).

All subjects were in good health condition confirmed by a normal medical history, physical and clinical laboratory examinations, vital signs measurement, a 12-lead electrocardiogram (ECG) assessment, and serology and drug of abuse tests. Subjects were excluded if they took an investigational medicinal product in the previous 2 months prior to the first study drug administration or donated blood in the previous 2 months, those with a history or presence of alcohol abuse (>7 units of alcohol/day for females and >14 units of alcohol/day for males), those with intake of foods or beverages containing xanthines for 58 h prior to dosing, smokers, those with regular intake of any medication in the previous 14 days prior to dosing, those who were pregnant, or in the lactation period.

Prior to the study, the volunteers were informed about the nature, purpose, risks, and discomforts that could arise from their participation, and about their right to withdraw at any time. Subjects documented their willingness to participate by signing the informed consent form. The clinical trial protocol and information given to study subjects were approved by the Romanian Competent Authorities (NAMMD) and by the Institutional Ethics Committee of University of Medicine and Pharmacy “Iuliu Haţieganu”, Cluj-Napoca, Romania. The study was performed in accordance to the Declaration of Helsinki (revised version of Seoul 2008) and the provisions of Good Clinical Practice [9].

### 2.3. Safety

The subjects were under constant supervision after the first administration of the drug and during hospitalisation. Subjects were interrogated on the occurrence of adverse events at each admission, before each study drug administration, and at about 2, 8, and 24 h after dosing in each study period. Additionally, all the subjects were asked to report immediately any adverse event.

At screening, blood pressure (systolic and diastolic) in the supine and standing position, heart rate (only in supine or sitting position), body temperature, and physical examination were assessed and recorded. An ECG was also performed. Vital signs (heart rate, blood pressure, and body temperature) of the subjects were monitored and recorded at each admission, before each study drug administration and approximately at about 2, 8, and 24 h after dosing in each study period. The vital signs, physical examination, and ECG were also performed at the end of the study.

For safety reasons, the laboratory tests performed at the screening visit (hematology, blood chemistry, and urinalysis) were repeated at the end of the study, except the serology tests.

### 2.4. Drug Products

The following formulations were used: rizatriptan 10 mg orodispersible tablets manufactured by Laboratorios Lesvi S.L., Sant Joan Despí, Spain, as the test product, and Maxalt^®^ Max 10 mg oral lyophilisates manufactured by Frosst Iberica S.A., Alcalá de Henares, Spain, as the reference product.

### 2.5. Analysis of Plasma Samples

Concentrations of rizatriptan in plasma samples were analysed using a validated method in compliance with FDA (Food and Drug Administration) Guidance [10]. The method involved a solid-phase extraction procedure followed by HPLC (high-performance liquid chromatography) with MS/MS (tandem mass spectrometry) detection. Calibration curves were linear in the range of 100.5–40,200.0 pg/mL with coefficients of correlation ≥0.9979. The validated stability period of the samples covered extensively the period between the first blood draw and completion of the analytical determination. The study was conducted in compliance with the Principles of Good Laboratory Practice [11].

### 2.6. Pharmacokinetic and Statistical Analysis

The pharmacokinetic parameters were estimated according to a non-compartmental method and were calculated individually for each subject from rizatriptan levels in plasma using Phoenix^®^ WinNonlin^®^ version 6.2 software (Pharsight, CA, USA). The pharmacokinetic analysis was done using the actual times of blood draws, and concentration values below the limit of quantification were set to zero for the analysis.

For the purpose of bioequivalence analysis, the primary pharmacokinetic parameters were AUC_0-t_ (area under the plasma concentration-time curve from zero to the last measurable concentration) calculated according to the linear trapezoidal rule and C_max_ (maximum plasma concentration) observed directly from experimental data. Other evaluated pharmacokinetic parameters were AUC_0-∞_ (area under the plasma concentration-time curve from zero to infinity) estimated by extrapolating to infinity AUC_0-t_, t_max_ (time to reach the maximal plasma concentration), and t_1/2_ (terminal half-life time).

The sample size was determined by taking into account published data [12] that showed a maximum coefficient of variation around 24%, derived from the residual variability obtained in the analysis of variance (ANOVA) after log-transformation for C_max_. According to the approach of Zhang [13], a total of 36 volunteers were considered sufficient to achieve a power of 90% with an alpha level protection of 0.05, assuming the a priori maximum difference of 5% between formulations. Finally, it was estimated that 40 subjects should be enough to meet the 80%–125% bioequivalence range with a statistical power of at least 90%, to take into account the possibility of observing dropouts/withdrawals and possible variations about the estimated intra-subject CV (coefficient of variation). 

The bioequivalence assessment was done using a parametric approximation for AUC_0-t_ and C_max_ after logarithmic transformation. For each parameter, the confidence interval for the difference between formulations on the log-transformed scale is obtained from the ANOVA (analysis of variance) model. This confidence interval is then back-transformed to obtain the desired confidence interval for the ratio on the original scale. The software used was Phoenix^®^ WinNonlin^®^ version 6.2. The statistical significance was established at *p* ≤ 0.05 for all statistical tests. The t_max_ was analysed nonparametrically by means of the Friedman rank sum test. 

Bioequivalence assessment was based on a predefined acceptance criterion of 80%–125% for the 90% confidence interval for the ratio of the test and reference products for the log-transformed data of AUC_0-t_ and C_max_. 

An ANOVA was performed for the primary parameters estimated in order to evaluate formulation, sequence, and period as fixed effects.

## 3. Results and Discussion

### 3.1. Study Population

Forty volunteers were included (20 males and 20 females), randomized and dosed into this clinical trial. One subject dropped out of the study after the first period due to personal reasons; hence 39 completed the crossover design receiving a single dose of both formulations and were included in the pharmacokinetic analysis. The safety analysis included the 40 subjects randomized and dosed.

### 3.2. Safety

No clinically significant abnormalities were found regarding physical examinations, vital sign measurements, ECG (electrocardiogram), and laboratory evaluations.

A total of 20 treatment-emergent adverse events occurred during the study, five after the administration of the test treatment, six after the administration of the reference treatment, and nine during the follow-up period.

The most frequently reported adverse events were headache (four cases) and leukocyturia (four cases). Most of the adverse events, 19 of 20, were of mild intensity. Six of the 20 adverse events were assessed as being related to the study medication, four occurred under the test treatment (headache and nausea), and two under the reference treatment (headache and nausea).

No serious adverse events occurred during the study. The safety profile of both formulations was consistent with the summary of product characteristics [8].

### 3.3. Drug Concentrations

Table 1 presents the arithmetic mean, standard deviation, coefficient of variation, median, minimum and maximum values for C_max_, AUC_0-t_ (area under the plasma concentration-time curve from zero to the last measurable concentration)_,_ AUC_0-∞_ (area under the plasma concentration-time curve from zero to infinity), t_max_ and t_1/2_ (terminal half-life time) tests, and reference parameters. The mean AUC_0-t_ value of the test formulation was 76,925.49 pg·h/mL (standard deviation (SD) 25,819.29 pg·h/mL) and the mean AUC_0-t_ of the reference formulation was 75,548.71 pg·h/mL (SD 22,210.48 pg·h/mL). C_max_ was 26,874.64 ± 11,441.15 pg/mL (mean ± SD) for the test formulation and 25,944.23 ± 9503.32 pg/mL for the reference one. Arithmetic means for all test and reference parameters are presented by gender too. 

**Figure 1:**
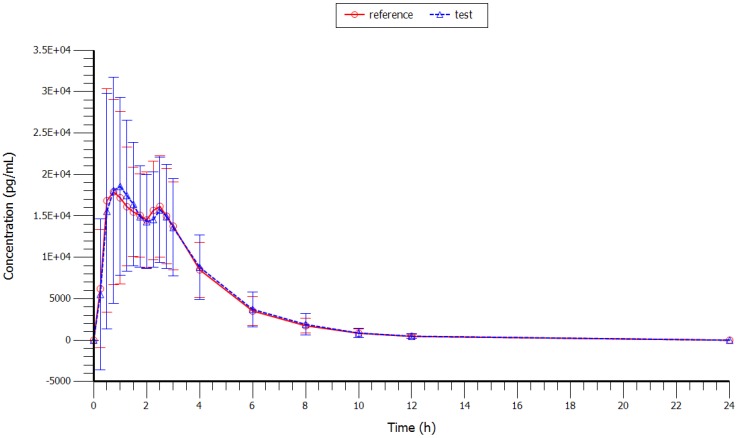
Linear profile of the mean concentrations vs. time curve of rizatriptan 10 mg after oral administration of a single dose of the test and reference formulations to 39 healthy volunteers.

**Figure 2:**
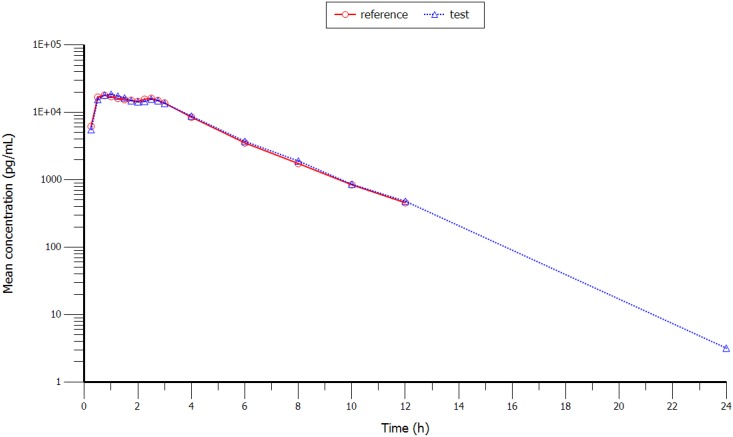
Semi-log profile of the mean concentration vs. time curve of rizatriptan 10 mg after oral administration of a single dose of the test and reference formulation**s** to 39 healthy volunteers.

Mean plasma concentrations (test and reference formulations) over the 24 h sampling period after administration are shown in Figure 1 (with and without SD) and in Figure 2 (semi-log scale). Plasma profiles were very similar for the test and reference formulations. The mean value of extrapolated AUC for both formulations was well below 20%.

### 3.4. Bioequivalence Evaluation

The 90% confidence interval (CI) for the ratio on the original scale (back-transformed from the 90% CI obtained for the difference between formulations on the log-transformed scale) for primary variables (AUC_0-t_ and C_max_) laid between the predefined range of 80%–125%. Results are presented in Table 2.

No period, no formulation, and no sequence effects were found in the ANOVA analysis for the primary pharmacokinetic parameters. The Friedman rank sum test performed on un-transformed data of t_max_ did not detect a statistically significant difference between the means.

### 3.5. Discussion and Conclusions

Based upon a 90% CI for the ratio of geometric LSmeans (least-squares means) (test/reference) of logarithmically transformed AUC_0-t_ and C_max_, the conclusion of bioequivalence can be made for the two orodispersible formulations of rizatriptan 10 mg (test and reference). Based on the study data, it can be confirmed that drug exposure between the reference and test formulations is equivalent. 

Both formulations were well-tolerated and no relevant differences in safety profiles between them were found.

In conclusion, considering that the observed plasma concentrations of the test and reference formulations are essentially similar as well as the safety data observed in this study, the generic formulation developed by Laboratorios Lesvi, S.L. is considered bioequivalent to the reference formulation and is expected to produce the same therapeutic response.

## Figures and Tables

**Table 1:** Un-transformed descriptive data of pharmacokinetic parameters for the test and reference formulations of rizatriptan 10 mg.

**TEST *n* = 39**	**C_max_ (pg/mL)**		**AUC_0-t_ (pg·h/mL)**		**AUC_0-∞_ (pg·h/mL)**		**t_max_ (h)**		**t_1/2_ (h)**	
**Arithmetic mean**	26,874.64		76,925.9		78,172.25		1.33		1.92	
	M = 20,366.34	F = 33,057.53	M = 61,683.85	F = 91,405.04	M = 62,840.23	F = 92,737.66	M = 1.54	F = 1.14	M = 2.07	F = 1.78
**SD**	11,441.15		25,819.29		26,047.53		0.85		0.35	
**CV (%)**	42.57		33.56		33.32		64.00		17.96	
**Median**	25,901.11		72,163.23		75,312.94		1.00		1.82	
**Min.**	11,371.00		36,192.66		37,010.46		0.25		1.42	
**Max.**	55,111.41		134,248.17		134,825.75		3.00		3.22	
**REFERENCE *n* = 39**	**C_max_ (pg/mL)**		**AUC_0-t_ (pg·h/mL)**		**AUC_0-∞_ (pg·h/mL)**		**t_max_ (h)**		**t_1/2_ (h)**	
**Arithmetic mean**	25944.23		75,548.71		76,850.48		1.29		1.90	
	M = 20,842.95	F = 30,790.44	M = 61,855.75	F = 88,557.02	M = 63,080.36	F = 89,932.08	M = 1.26	F = 1.33	M = 2.00	F = 1.81
**SD**	9503.32		22,210.48		22,716.91		0.78		0.28	
**CV (%)**	36.63		29.40		29.56		60.06		14.58	
**Median**	24,471.16		71,601.68		72,573.35		1.00		1.90	
**Min.**	8708.50		36,887.53		37,801.38		0.50		1.40	
**Max.**	52,634.86		123,674.07		126,953.51		2.50		2.77	

M = Males (*n* = 19). F = Females (*n* = 20). CV: coefficient of variation; AUC: area under the plasma concentration-time curve; t_max_: time to reach maximum plasma concentration; t_1/2_: terminal half-life time; SD: standard deviation.

**Table 2:** Bioequivalence assessment summary for rizatriptan of rizatriptan 10 mg.

Parameter (Log-Transformed Data)	Geometric LSMean		T/R Ratio	90% CI
	Test	Reference	(%)	(%)
C_max_ (pg/mL)	24,462.20	24,307.17	100.64	91.98–110.11
AUC_0-t_ (pg·h/mL)	72,719.66	72,342.60	100.52	97.07–104.09

LS Mean: Least-squares mean; T/R: Test/Reference; CI: Confidence interval.
